# Effects of Equal Volume Heavy-Resistance Strength Training Versus Strength Endurance Training on Physical Fitness and Sport-Specific Performance in Young Elite Female Rowers

**DOI:** 10.3389/fphys.2020.00888

**Published:** 2020-07-21

**Authors:** Dirk Thiele, Olaf Prieske, Melanie Lesinski, Urs Granacher

**Affiliations:** ^1^Division of Training and Movement Sciences, Research Focus Cognitive Sciences, University of Potsdam, Potsdam, Germany; ^2^Division of Exercise and Movement, University of Applied Sciences for Sports and Management Potsdam, Potsdam, Germany

**Keywords:** concurrent training, plyometric training, on-water performance, race time, oarsmen

## Abstract

Strength training is an important means for performance development in young rowers. The purpose of this study was to examine the effects of a 9-week equal volume heavy-resistance strength training (HRST) versus strength endurance training (SET) in addition to regular rowing training on primary (e.g., maximal strength/power) and secondary outcomes (e.g., balance) in young rowers. Twenty-six female elite adolescent rowers were assigned to an HRST (*n* = 12; age: 13.2 ± 0.5 yrs; maturity-offset: +2.0 ± 0.5 yrs) or a SET group (*n* = 14; age: 13.1 ± 0.5 yrs; maturity-offset: +2.1 ± 0.5 yrs). HRST and SET comprised lower- (i.e., leg press/knee flexion/extension), upper-limbs (i.e., bench press/pull; lat-pull down), and complex exercises (i.e., rowing ergometer). HRST performed four sets with 12 repetitions per set at an intensity of 75–95% of the one-repetition maximum (1-RM). SET conducted four sets with 30 repetitions per set at 50–60% of the 1-RM. Training volume was matched for overall repetitions × intensity × training per week. Pre-post training, tests were performed for the assessment of primary [i.e., maximal strength (e.g., bench pull/knee flexion/extension 1-RM/isometric handgrip test), muscle power (e.g., medicine-ball push test, triple hop, drop jump, and countermovement jump), anaerobic endurance (400-m run), sport-specific performance (700-m rowing ergometer trial)] and secondary outcomes [dynamic balance (Y-balance test), change-of-direction (CoD) speed (multistage shuttle-run test)]. Adherence rate was >87% and one athlete of each group dropped out. Overall, 24 athletes completed the study and no test or training-related injuries occurred. Significant group × time interactions were observed for maximal strength, muscle power, anaerobic endurance, CoD speed, and sport-specific performance (*p* ≤ 0.05; 0.45 ≤ *d* ≤ 1.11). *Post hoc* analyses indicated larger gains in maximal strength and muscle power following HRST (*p* ≤ 0.05; 1.81 ≤ *d* ≤ 3.58) compared with SET (*p* ≤ 0.05; 1.04 ≤ *d* ≤ 2.30). Furthermore, SET (*p* ≤ 0.01; *d* = 2.08) resulted in larger gains in sport-specific performance compared with HRST (*p* < 0.05; *d* = 1.3). Only HRST produced significant pre-post improvements for anaerobic endurance and CoD speed (*p* ≤ 0.05; 1.84 ≤ *d* ≤ 4.76). In conclusion, HRST in addition to regular rowing training was more effective than SET to improve selected measures of physical fitness (i.e., maximal strength, muscle power, anaerobic endurance, and CoD speed) and SET was more effective than HRST to enhance sport-specific performance gains in female elite young rowers.

## Introduction

Competitive rowing is a traditional Olympic sport with high demands on several components of physical fitness such as anaerobic endurance, strength endurance, and maximal strength (Gee et al., 2011; Akça, 2014). For instance, lower and upper limbs strength endurance (i.e., leg press 30- repetition maximum, RM and seated arm pulling 60-RM) were significantly associated with sport-specific performance (2,000-m rowing ergometer trial) in elite rowers (−0.66 ≤ *r* = ≤ −0.60) (Jürimäe et al., 2010; Lawton et al., 2013). Even though no cause-effect relations were established, these findings imply that rowers with higher levels of physical fitness (e.g., strength endurance) are those athletes with better rowing performances or vice versa. Gains in physical fitness may therefore translate to rowing-specific performance improvements. There is ample evidence indicating that strength training is an effective means to enhance muscle strength (i.e., maximal strength and strength endurance) as well as sport-specific performances in healthy individuals, irrespective of age, sex, and training status (Rhea et al., 2003; Sedano et al., 2013; Lesinski et al., 2016; Schoenfeld et al., 2017). In rowing, strength endurance training (SET) programs have been proposed as standard strengthening routines (Bompa and Buzzichelli, 2015). Moreover, Lawton et al. (2011) suggested beneficial effects of SET as well as heavy-resistance strength training (HRST) on rowing-specific performance. For young athletes irrespective of the practiced sport, there is evidence that HRST is effective in improving physical fitness and sport-specific performances (Lesinski et al., 2016). The computation of dose-response relations showed that strength training programs with fewer repetitions (6–8 repetitions per set) and higher intensities (i.e., 80% 1-RM) appear to be more effective to improve measures of muscle strength in young adolescent athletes compared with programs characterized by low intensity and a high number of repetitions (Lesinski et al., 2016). In another study, Ebben et al. (2004) investigated the effects of a periodized 8-week HRST versus SET in addition to the regular sport-specific training on rowing performance (e.g., 2,000-m ergometer time) in female recreational and elite rowers aged 20 years. Interestingly, non-significant differences were found between HRST and SET effects on sport-specific performance. A recently published systematic review with meta-analysis on the effects of strength training on lower limb maximal strength and rowing performance in rowers of different expertise levels revealed small-sized effects of strength training on both performance outcomes, irrespective of the training type (i.e., HRST vs SET) and rowers’ expertise level (e.g., recreational, sub-elite, and elite) (Thiele et al., 2020). To the best of our knowledge, there is no study available that directly contrasted the effects of HRST versus SET on components of physical fitness and sport-specific performance in young rowers.

Thus, the purpose of this controlled trial was to examine the effects of equal volume HRST versus SET in addition to regular rowing training on primary (i.e., maximal strength, muscle power, anaerobic endurance, and sport-specific performance) and secondary [i.e., dynamic balance, change-of-direction (CoD) speed] outcomes in young female rowers.

With reference to the relevant literature, we hypothesized that both training programs (HRST and SET) in addition to regular rowing training induce gains in primary and secondary outcome measures (Lesinski et al., 2016; Thiele et al., 2020), with potentially larger improvements following HRST (Sedano et al., 2013; Lesinski et al., 2016; Schoenfeld et al., 2017).

## Materials and Methods

### Experimental Protocol

We studied adaptations following 9 weeks of HRST versus SET in addition to regular rowing training in young elite female rowers using a two-group repeated measures design. Our primary outcomes were maximal strength, muscle power, anaerobic endurance (i.e., 400 m run), and sport-specific performance (i.e., 700-m rowing ergometer time trail). Secondary outcomes included dynamic balance and CoD speed. Pre-and post-tests were carried out at the same time of day using the same test sequence. The test period lasted 5 days for baseline and post-tests. All rowers were familiarized with the test procedure prior to testing. Before testing, a standardized 20-min warm-up was performed consisting of dynamic stretching, jumping, running, and agility/CoD drills.

### Participants

With reference to the study of Sedano et al. (2013), we computed an *a priori* power analysis using G × Power (Version 3.1.9.2, University of Kiel, Kiel, Germany) and the *F* test family (Faul et al., 2007) with an assumed Type I error of 0.05 and a type II error rate of 0.20 (80% actual statistical power) for the effects of strength training on proxies of sport-specific performance. The analysis revealed that 24 participants would be sufficient to observe a large-sized group × time interaction for sport-specific performance. Due to potential dropouts (e.g., injuries and illness), 26 young elite female rowers were enrolled in this study and two athletes dropped out due to personal reasons. Twelve rowers with 2 years of rowing experience (elite) and competition success on a national level participated in this study. The other 14 rowers were on regional competitive level. Fourteen rowers were assigned to the SET group and 12 rowers attended the HRST group. We acknowledge that due to the sequential study design, the current study was a non-randomized controlled trial. The expertise level was equally distributed across the two experimental groups. SET group conducted their training between January and March 2016 while HRST group performed their program from January to March 2017. This sequential study design was implemented due to the limited number of rowers per cohort. Of note, all participants attended the elite sport school in Potsdam, Germany. Based on systematic talent identification programs, only a limited number of young rowers is selected each year. These circumstances were responsible for the sequential study design. Participants’ characteristics are displayed in Table 1. The maturity status was determined according to the maturity offset method as introduced by Mirwald et al. (2002). The training program was similar for all participating athletes. All rowers participated in regular physical education classes (five lessons/week, 90 min each) in addition to regular rowing training (two training sessions/week, 90 min each). In total, a training volume of ten hours per week was scheduled. All athletes were experienced in performing strength training using exercises like those applied during the study. The participants performed rowing ergometer training prior to and during the study as part of their regular training. At the beginning of the study, all participants and their legal representatives were informed about the benefits and risks of the investigation and were kindly asked to sign a written informed consent. All individuals were familiarized with the experimental protocol. This study was conducted in accordance with the latest version of the declaration of Helsinki. The protocol was approved by the local ethical commission (University of Potsdam: submission No. 5/2014).

**TABLE 1:** Characteristics of the study participants.

**Training**	**SET *n* = 13**				**HRST *n* = 11**				**Total *n* = 24**			
	**Means Pre**	**Standard deviations**	**Means Post**	**Standard deviations**	**Means Pre**	**Standard deviations**	**Means Post**	**Standard deviations**	**Means Pre**	**Standard deviations**	**Means Post**	**Standard deviations**
Age	13.1	0.5	13.3	0.5	13.2	0.5	13.4	0.5	13.2	0.5	13.4	0.5
Standing body height (cm)	175.4	4.0	175.8	3.9	174.2	4.9	175.2	4.9	174.8	4.4	175.5	4.3
Sitting body height (cm)	91.1	3.5	91.4	3.3	90.9	2.7	91.1	3.2	91.0	3.1	91.3	3.2
Body mass (kg)	64.3	7.6	65.4	6.8	63.2	6.0	63.8	5.6	63.8	6.8	64.6	6.2
BMI	20.9	2.1	21.0	2.0	20.9	1.7	20.8	1.6	20.9	1.9	20.9	1.8
PHV	11.1	0.3	11.2	0.4	11.1	0.3	11.2	0.3	11.1	0.3	11.2	0.4
Maturity offset	2.1	0.5	2.2	0.6	2.0	0.5	2.2	0.5	2.0	0.5	2.2	0.5

*BMI, body mass index; HRST, heavy-resistance strength training; PHV, peak velocity high; SET, strength endurance training.*

### Intervention

Testing and training were conducted during the preparation phase from January to March 2016 and 2017, respectively. Athletes from both experimental groups performed identical strength exercises for 9 weeks as described in Table 2. Previous studies have shown that this time period is long enough to elicit significant gains in physical fitness and sport-specific performance (Izquierdo-Gabarren et al., 2010; Lesinski et al., 2015; Thiele et al., 2020). Two strength training sessions were scheduled per week. While SET group performed a traditional strength endurance program with 30 repetitions per set at an intensity of 50–60% of the 1-RM and a 1 min rest between sets (Fleck and Kraemer, 2004), HRST group conducted four sets with 12 repetitions per set at an intensity of 75–95% of the 1-RM and a 2 min rest between sets. Training intensity (i.e., external load) was progressively adjusted according to the internal training load by evaluating session rating of perceived exertion (RPE) on a 10-point Likert scale based on the computed session RPE (Foster et al., 2001). More specifically, for each strength exercise, the individually applied external load had to correspond to an exercise RPE range of 8 (“extreme exhaustion”) to 9 (“maximal exhaustion”). Furthermore, after 4 weeks of training, 1-RM testing was performed for all strength exercises to adjust the training loads. Training volume was similar between groups. This was realized by adjusting the volume for repetitions × intensity × training per week (i.e., tonnage) (Table 3). During the intervention period, all participants continued their on-land conditioning program consisting of 7.5 h per week of stretching, coordination training, running exercises and HRST or SET. In addition, they performed rowing ergometer training for 2–3 h per week in preparation of the competitive season. The conditioning and ergometer training programs were similar between groups. Training differed with regards to the applied strength training programs (HRST vs SET). For both experimental groups, strength training was always conducted prior to endurance training. If applied on the same day, a rest of 7 h was granted between strength and endurance training (Sousa et al., 2020).

**TABLE 2:** Exercises conducted during strength training.

**Heavy-resistance strength Training (two sessions per week)**	**Strength endurance training (one session per week)**	**Strength endurance training (one session per week)**
Bench pull	Bench pull	Bench pull
Leg press	Leg press	Knee flexion
Rowing ergometer	Lat pull down	Bench press
Lat pull down	Rowing ergometer	Knee extension
Knee flexion		
Bench press		
Knee extension		

**TABLE 3:** Training characteristics of the heavy-resistance strength training (HRST) versus strength endurance training (SET).

**Methods**	**Training per week**	**Intensity**	**Repetitions**	**Sets**	**Number of exercises**	**Overall repetitions**	**Overall rep × intensity × training per week**
HRST	2	75–95%	12	4	7	336	504–604
SET	2	50–60%	30	4	4	480	480–576

*Rep, repetitions.*

### Assessment of Anthropometric Characteristics

Participants’ anthropometric characteristics were assessed using a mobile body height measurement device (Stadiometer seca213, seca, Hamburg, Germany) and measuring tape for standing body height (m), sitting height (cm), and leg length (cm). Body composition (i.e., total and segmental lean body- and fat-mass) was analyzed using a bioelectrical impedance analysis system (InBody 720, Biospace, Seoul, South Korea). Tests for body composition were always conducted in the morning in a fasted state using a standardized protocol^1^.

### Physical Fitness Tests

During talent development, a focus should be laid on exercising different components of physical fitness to establish a broad foundation of physical qualities for subsequent sport-specific performances (Lloyd et al., 2015). Accordingly, we selected a large number of physical fitness tests ranging from strength and endurance to balance and CoD speed tests to evaluate the effectiveness of the two strength training programs.

#### Primary Outcomes

##### Assessment of maximal strength

The following tests were performed in accordance with the ACSM’s guidelines for 1-RM testing (e.g., bench pull, leg press, knee extension, and knee flexion) (Pescatello, 2014). Bench pull (*r* = −0.75, *p* < 0.01) and leg-press (*r* = −0.75, *p* < 0.01) were identified as two important maximal strength tests to predict sport-specific performance in college rowers aged 20.2 ± 1.2 years (Akça, 2014). Bench pull performance was tested in prone position on a bench. The examiner visually inspected whether the arms were straight as participants grabbed the barbell. The athletes performed a concentric arm flexion beginning from the extended position. The end position was determined by touching the bench (Akça, 2014). The leg-press exercise was performed on a 45° inclined leg-press machine. The feet (shoulder-width apart, out-toeing <30°) were placed in the center of the leg-press plate. Knee flexion was constant and limited to 90° (Jürimäe et al., 2010). Knee extension was tested during sitting position on the machine with the legs under the pad (feet pointed forward, starting position 90°) and the hands holding on to the sidebars. The knees were extended until they reached a 180° position to ensure that the remaining body segments were fixed and stable on the seat. For knee flexion, participants were in prone position with their knees fully extended. A load was attached to the back of the calf. Participants were instructed to bring the lower leg closer to the thigh by bending the knee. As a general fitness marker, isometric handgrip strength was assessed (Jamar, Warrenville, IL, United States). For this purpose, participants were placed on a chair with the dynamometer in the dominant hand and the elbow flexed at 90°. Hand dominance was assessed using the lateral preference inventory questionnaire (Coren, 1993). Participants were asked to press the dynamometer as forcefully as possible for 3 s. Three trials were carried-out and the best performance was used for further data processing.

##### Assessment of muscle power

To assess lower limb muscle power, participants performed maximal vertical countermovement jumps (CMJs) and drop jumps (DJs) on a three-dimensional force plate (type 9286AA; Kistler, Winterthur, Switzerland). The vertical ground reaction force was sampled at 1,000 Hz. Three CMJ and DJ tests trials were conducted with a resting period of 30 s between jumps and a 1 min recovery between CMJ and DJ tests. Drop height was 40 cm during the performance of DJs. The best trial in terms of best maximal jump height was taken for further data analysis. Jump height (e.g., DJ/CMJ height) was calculated according to the following formula: jump height = 1/8×*g*×*t*^2^, where *g* is the acceleration due to gravity and *t* is the flight time (Prieske et al., 2013). Additionally, we recorded ground contact time during the performance of DJs and computed the reactive strength index by dividing jump height by ground contact time (i.e., DJ RSI) (Prieske et al., 2019). For lower and upper limbs muscle power, the triple hop jump test and the medicine ball throw test were applied. The triple hop jump test started in the step position with one foot on the starting line. Two consecutive jumps on one leg were completed with a final standing long jump. The medicine ball throw test was performed with a 3 kg medicine ball starting in a step position. All participants performed three attempts and the best trial was used for statistical analysis.

##### Assessment of anaerobic endurance

Anaerobic endurance was measured by performing an all-out 400-m run. The test was conducted on a 200-m indoor track to avoid bias due to weather conditions. Time to complete the 400-m was used as the dependent variable for further analysis.

##### Assessment of sport-specific rowing performance

In this study, a 700-m all-out rowing ergometer test was applied using Concept II ergometers (Model D, Morrisville, Vermont, United States) with a drag factor of 120 to assess sport-specific performance. In the 12 year old age category, 500-m ergometer test distances are regularly applied at the elite sport school. As athletes move on to the 13-year old age category, ergo tests are realized over 1,000-m distances. Given that the difference between the 500 and the 1,000-m test is large, 700-m ergo tests are always performed at the beginning of the age category 13 to familiarize young rowers with the longer test distances. High test retest reliability and correlations for different distances on rowing ergometer were approved in different studies (Ingham et al., 2002; Soper and Hume, 2004; Maciejewski et al., 2016).

Stroke rate was not predefined, and athletes were expected to exert maximal effort for the duration of the entire test. The average power was recorded for further analysis. Athletes’ warm-up consisted of 10 min of mobilizing exercises, 15 min of running or cycling, and 15 min of submaximal rowing on the rowing ergometer.

#### Secondary Outcomes

There are primarily two pathways that are pursued to develop a gifted young child into a talented elite athlete. These are early specialization and diversification (Côté et al., 2009). While both pathways have proven to be successful in developing high performance athletes, more recent evidence has suggested that there is an increased risk with early specialization to sustain acute and/or overuse injuries which may ultimately lead to drop out from organized sports (DiFiori et al., 2014; Jayanthi et al., 2015). Diversification on the other hand has proven to be particularly successful with cgs (centimeters, grams, and seconds) sports in terms of developing successful elite athletes (Moesch et al., 2011). A premise of the diversification approach is to lay a foundation of physical fitness before developing sport-specific performance (Malina et al., 2010). In other words, fitness development precedes sport specialization. Accordingly, a broad foundation of physical fitness components should be trained and tested during the early stages of long-term athlete development, irrespective of the sport young athletes practicing at this stage.

Given that the enrolled athletes just started their sporting career at the elite sport school, we included these rather generic fitness tests that may not directly relate to rowing performance to lay a broad foundation of physical fitness.

##### Assessment of dynamic balance

Dynamic balance was assessed using the Y-balance-test (Plisky et al., 2006). Before the test started, participants’ left and right leg length were assessed. This was realized in supine lying position. Thereafter, the distance from the anterior superior iliac spine to the most distal aspect of the medial malleolus was measured. Further, participants practiced three trials per reach direction on each foot to get familiarized with the testing procedures. All trials were conducted barefoot. Participants were positioned in single-leg stance while reaching as far as possible with the contralateral leg in three different reach directions (i.e., anterior, posteromedial, and posterolateral). The start of the test was standardized by using the right foot placed at the center of the Y-balance-test tool (Move2Perform, Evansville, IN, United States) and the left leg reaching three times in anterior direction as far as possible. Thereafter, the left foot was placed at the center of the grid and the right leg reached in anterior direction. Thereafter, the same test procedure was realized for the posteromedial and the posterolateral reach direction (positioned 135° from the anterior scale). The tester manually measured the distance from the scale of the tool. A composite score was calculated and taken as dependent variable for further data analyses using the following formula: composite score = [(maximum anterior reach distance + maximum posteromedial reach distance + maximum posterolateral reach distance)/(leg length × 3)] × 100 (Filipa et al., 2010).

##### Assessment of change-of-direction speed

A multi-stage shuttle run test (“Japan-Test”) was implemented to gather performance in CoD speed performance. Athletes had to shuttle between a 4.5-m range by using side steps for five times and touch the lines with their hands. The best out of two trials was used for further analysis. Time to complete the Japan-Test was used as the dependent variable for further analysis.

### Statistical Analyses

Data are presented as group mean values and standard deviations. Normal distribution of data was examined and confirmed using the Shapiro–Wilk test. To evaluate the effects of training, a 2 (group: HRST, SET) × 2 (time: pre, post) ANCOVA with repeated measures on time was computed. As a covariate, pre-post changes in standing body height were included in the statistical model. If “group × time” interactions reached the level of significance, group-specific *post hoc* tests (i.e., paired *t*-tests) were conducted to identify the comparisons that were statistically significant. Additionally, effect sizes were calculated by converting partial eta-squared to Cohen’s *d* to indicate whether a statistical difference is a difference of practical concern. According to Cohen (1988), the magnitude of effect sizes can be classified as small (0.2 ≤ *d* < 0.5), medium (0.5 ≤ *d* < 0.8), and large (*d* ≥ 0.8). The significance level was set at α < 0.05. To assess test-retest reliability, cut-offs for ICC values ≥0.80 were rated “acceptable” (Hopkins, 2000). All analyses were performed using Statistical Package for Social Sciences (SPSS) version 25.0.

## Results

All participants received treatment conditions as allocated. One participant from HRST group left the elite sport school for personal reasons and another athlete from SET group was excluded due to an injury not related to the intervention. These two rowers were not included in the final analysis. Thus, 24 young female athletes completed the intervention period, and none reported any test- or training-related injuries. All included tests revealed high test-retest reliability (Table 4). Group-specific mean pre- and post-test values for primary and secondary outcomes are presented in Table 5. No significant between-group baseline differences were found for most fitness outcomes (all *p* > 0.05), except for leg-press (*p* = 0.032), and knee flexion (*p* = 0.007) exercises. In terms of participants’ characteristics, the statistical analysis revealed a significant group × time interaction for standing body height (*p* ≤ 0.01, *d* = 0.62) with significantly larger body height increases following HRST only (*p* = 0.001, *d* = 1.65). Table 6 illustrates main effects for group, time, and group × time interactions.

**TABLE 4:** Specification of the intraclass correlation coefficients (ICC) for primary and secondary outcomes either from the literature or unpublished data from our laboratory.

	**Outcomes**	**Test**	**ICC**	**Reference/own data**
	Anthropometrics	Body composition	0.97–0.99	Ling et al. (2011)
Primary outcomes	Maximal strength	Bench pull	0.99	*n* = 9 [own data]
		Leg press	0.96	*n* = 7 [own data]
		Knee extension	0.95	*n* = 10 [own data]
		Knee flexion	0.85	*n* = 10 [own data]
		Isometric handgrip strength	0.98	Bellace et al. (2000)
	Muscle power	Medicine ball push test	0.95	*n* = 7 [own data]
		Triple hop jump test	0.90	*n* = 7 [own data]
		DJ	0.95	Tenelsen et al. (2019)
		CMJ	0.98	Markovic et al. (2004)
	Anaerobic endurance	400-m run	0.96	*n* = 10 [own data]
	Sport-specific performance	700-m ergometer trial	0.93–0.99	Soper and Hume (2004)
Secondary outcomes	Dynamic balance	Y-balance	0.89–0.93	Plisky et al. (2006)
	Change of direction speed	Multistage shuttle run	0.85	*n* = 7 [own data]

*CMJ, countermovement jump; DJ, drop jump; ICC, intraclass correlation coefficient.*

**TABLE 5:** Characterization of pre- and post-test data for all outcome variables for heavy-resistance strength training (HRST) and strength endurance training (SET) group.

			**HRST**					**SET**				
	**Outcome**	**Variables**	**Pre**		**Post**		**Δ (%)**	**Pre**		**Post**		**Δ (%)**
			**Mean**	**SD**	**Mean**	**SD**		**Mean**	**SD**	**Mean**	**SD**	
Primary outcomes	Maximal strength	Bench pull	40.2	4.5	45.7	4.5	13.5	41.5	6	44.2	6.2	6.5
		Leg press	67	16.2	89.5	22.4	33.6	54.6	15.9	75.4	24.4	38.2
		Knee extension	49	7.4	61.5	9.4	25.5	53.1	8.8	56.5	11.1	6.5
		Knee flexion	37.2	2.6	43.3	7.1	16.4	46.3	9.1	47.9	6.9	3.6
		Isometric handgrip strength	29.7	4.3	32.1	4.7	8.1	31.7	6	31.5	6.3	−0.4
	Muscle power	Medicine ball push test	6.7	0.6	7.8	0.8	16	7.4	1.1	6.8	0.9	−8.4
		Triple hop jump test	5.2	0.2	5.7	0.4	10.4	5.5	0.5	5.5	0.5	0.2
		DJ height	21.5	2.6	19.2	4.1	−10.9	20.9	3.2	19.2	3.7	−8.2
		DJ RSI	1.01	0.17	0.91	0.22	−9.9	0.89	0.22	0.77	0.2	−13.5
		DJ contact time	214.5	19.2	211.5	17.7	−1.4	218.5	19.5	234.9	23.1	7.5
		CMJ height	23.5	2.8	22	3	−6.3	23	3.2	22.2	3.1	−3.4
	Anaerobic endurance	400−m run	89	6.5	77.6	6.6	−12.8	81.6	8.2	80.5	8	−1.5
	Sport-specific performance	700-m ergometer trial	228.6	29.5	241.2	31.7	5.5	220.6	36.1	235.1	33.3	6.6
Secondary outcomes	Dynamic balance	Y-balance	102.8	4.4	104.4	4.6	1.6	100.6	5.0	102.7	5.7	2,1
	Change-of-direction speed	Multistage shuttle run	8.5	0.3	8.1	0.4	−5.2	8.6	0.7	8.5	0.5	−1.1

*CMJ, countermovement jump; DJ, drop jump; RSI, reactive strength index; SD, standard deviation.*

**TABLE 6:** Main and interaction effects following heavy-resistance strength training (HRST) and strength endurance training (SET) in young female rowers.

	**Outcome**	**Variables**	**Time**		**Group**		**Interaction**	
		
			***p***	***d***	***p***	***d***	***p***	***d***
Primary outcomes	Maximal strength	Bench pull	0.001	2.15	0.571	0	0.002	0.60
		Leg press	0.001	0.59	0.066	0.167	0.71	0.01
		Knee extension	0.001	0.87	0.906	0	0.021	0.49
		Knee flexion	0.058	0.38	0.067	0.52	0.154	0.22
		Isometric handgrip strength	0.06	0.29	0.863	0.01	0.186	0.16
	Muscle power	Medicine ball push test	0.376	0.07	0.797	0.2	0.001	1.11
		Triple hop jump test	0.045	0.42	0.636	0.01	0.036	0.46
		DJ height	0.015	0.52	0.827	0	0.767	0.01
		DJ RSI	0.028	0.43	0.195	0.16	0.684	0.02
		DJ contact time	0.105	0.24	0.187	0.25	0.007	0.63
		CMJ height	0.243	0.12	0.643	0	0.522	0.04
	Anaerobic endurance	400-m run	0.001	0.92	0.498	0.06	0.001	0.76
	Sport-specific performance	700-m ergometer trial	0.003	0.95	0.491	0.06	0.006	0.85
Secondary outcomes	Dynamic balance	Y-balance test	0.104	0.23	0.579	0.03	0.46	0.03
	Change-of-direction speed	Multistage shuttle run	0.01	0.59	0.368	0.08	0.028	0.45

*CMJ, countermovement; *d*, effect size; DJ, drop jump; RSI, reactive strength index; SD, standard deviation.*

### Effects of HRST Versus SET on Primary Outcomes

**FIGURE 1:**
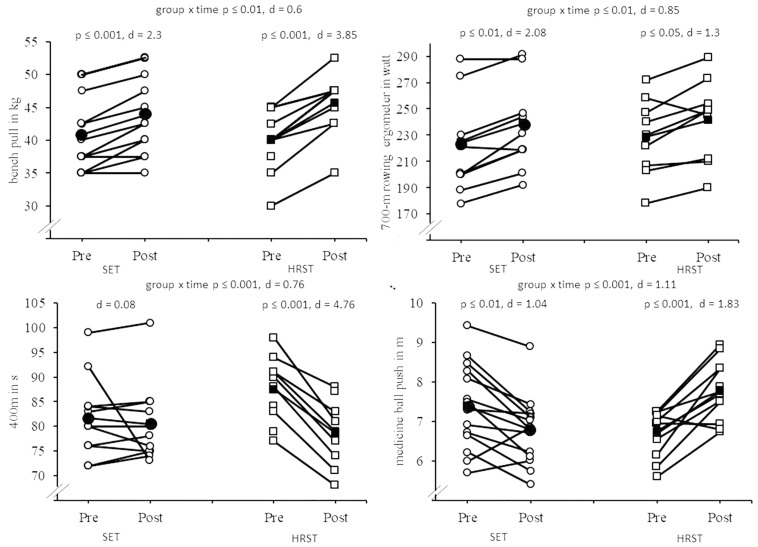
**(A–D)** Individual and mean pre- and post-test data for **(A)** bench pull, **(B)** 700-m rowing ergometer time trial, **(C)** 400-m run and **(D)** medicine ball push test according to the intervention group (HRST, heavy-resistance strength training; SET, strength endurance training). Unfilled circles indicate individual data for SET, filled circles indicate mean data for SET. Unfilled squares indicate individual data for HRST, filled squares indicate mean data for HRST.

In terms of maximal strength, the statistical analyses indicated a significant group × time interaction for bench pull and knee extension (*p* ≤ 0.05, 0.49 ≤ *d* ≤ 0.6). *Post hoc* analyses revealed larger effect sizes following HRST for bench pull (Δ 13.5%, *p* ≤ 0.001, *d* = 3.58) and knee extension (Δ 25.5%, *p* < 0.01, *d* = 1.81) compared with SET (bench pull: Δ 6.5%, *p* ≤ 0.001, *d* = 2.3; knee extension: Δ 6.5%, *p* ≤ 0.01, *d* = 1.09; Figure 1).

In terms of muscle power, the statistical analysis revealed significant group × time interactions for DJ contact time, the triple hop jump test, and the medicine ball push test (*p* ≤ 0.01, 0.46 ≤ *d* ≤ 1.11). *Post hoc* tests indicated that HRST resulted in a significant increase in the medicine ball push test (Δ 16.0%, *p* ≤ 0.001, *d* = 1.83), while SET showed a performance decline (Δ−8.4%, *p* ≤ 0.01, *d* = 1.04) (Figure 1). Furthermore, HRST but not SET produced significant improvements in triple hop jump test performance (Δ 10.4%, *p* ≤ 0.05, *d* = 1.58).

In terms of anaerobic endurance, our analysis revealed a significant group × time interaction effect (*p* ≤ 0.001, *d* = 0.76) for the 400-m all-out run (Table 6). *Post hoc* tests showed a significant performance improvement for HRST (Δ 12.8%, *p* ≤ 0.001, *d* = 4.76) but not SET (Figure 1).

In terms of sport-specific rowing performance, the statistical analysis indicated a significant group × time interaction for the 700-m rowing ergometer trial (*p* ≤ 0.01, *d* = 0.85) (Table 6). *Post hoc* analyses revealed larger beneficial effects following SET e (Δ 6.6%, *p* ≤ 0.01, *d* = 2.08) compared with HRST (Δ 5.5%, *p* < 0.05, *d* = 1.3; Figure 1).

### Effects of HRST Versus SET on Secondary Outcomes

A significant group × time interaction (*p* ≤ 0.05, *d* = 0.45) was found for CoD speed (i.e., multistage shuttle run). The *post hoc* analysis revealed significant performance improvements following HRST (Δ−5.2%, *p* ≤ 0.01, *d* = 1.84).

No significant group × time interaction effect was identified for dynamic balance.

## Discussion

To the authors’ knowledge, this is the first controlled trial that examined the effects of equal volume HRST versus SET in addition to regular rowing training on proxies of physical fitness and sport-specific performance in young elite female rowers. The main findings of this study are: (i) HRST resulted in larger gains in maximal strength (i.e., 1-RM bench pull and knee extension), muscle power (i.e., medicine ball push and triple hop), anaerobic endurance (i.e., 400-m run), and CoD speed (i.e., multistage shuttle run) compared with SET; (ii) SET enhanced larger gains in sport-specific performance (i.e., 700-m rowing ergometer trial) compared with HRST; and (iii) HRST and SET were similar effective in improving measures of dynamic balance in young elite female rowers.

In accordance with our study hypothesis, the present findings indicate that HRST and SET induced significant small-to-large sized effects in primary outcomes and small-to-medium sized effects in secondary outcome measures. Thus, 9 weeks of strength training appears to be an effective means to improve maximal strength (e.g., bench pull), muscle power (e.g., medicine ball push), anaerobic endurance (e.g., 400-m run), sport-specific performance (e.g., 700-m rowing ergometer trial), dynamic balance (e.g., Y-balance), and CoD speed (e.g., multistage shuttle run) in young elite female rowers. These findings are in line with the scientific literature with regards to the effects of strength training on physical fitness in young athletes. In their systematic review with meta-analysis, Lesinski et al. (2016) reported significant and large-sized effects (0.8 ≤ standardized mean differences, SMD ≤ 1.09) of strength training on maximal strength (e.g., 1-RM) and muscle power (e.g., jump performance) as well as significant medium-sized effects (0.68 ≤ SMD ≤ 0.75) on sport-specific performance and CoD speed in young athletes (Lesinski et al., 2016).

Interestingly, we found that HRST induced larger gains in measures of maximal strength (i.e., 1-RM bench pull and knee extension) compared with SET in young elite female rowers. This finding is in accordance with the principle of training specificity. In other words, larger training-induced effects are found if the training program mimics the requirements of the tested outcome (Haff and Triplett, 2016). Lesinski et al. (2016) showed that strength training programs with fewer repetitions (6 to 8 repetitions per set) and higher intensities (80–89% of 1-RM) were more effective to improve maximal strength in young athletes compared with programs applying lower intensities and more repetitions. In our study, HRST comprised fewer repetitions (12) and higher intensities (75–95% of 1-RM) compared with SET (30 repetitions at 50–60% of 1-RM). Thus, our HRST program followed the reported dose-response relation for strength training with young athletes (Lesinski et al., 2016). Furthermore, in a recent systematic review and meta-analysis, Schoenfeld et al. (2017) included 21 studies and contrasted the effects of HRST versus SET on muscle strength in trained and untrained male and female adults. The authors found significantly larger gains in maximal strength following HRST (SMD = 1.69) compared with SET (SMD = 1.32). In this regard, Schoenfeld et al. (2017) suggested that HRST produced larger effects on maximal strength due to its specific demands to lift maximal loads which is in accordance with the principle of training specificity. In line with Schoenfeld’s argumentation, it can be hypothesized that the specific demands of HRST also resulted in larger improvements in maximal strength in young elite female rowers.

With regards to muscle power (e.g., medicine ball push and triple hop), our analyses revealed larger training-induced adaptations following HRST compared with SET in young elite female rowers. Of note, HRST represents an important element during periodized strength training because it lays the foundations for later power performances (Hoffman, 2002; Bompa and Buzzichelli, 2015; Behm et al., 2017). In fact, in a systematic review with meta-analysis including 107 studies, Behm et al. (2017) examined the effects of strength training (predominantly HRST) versus power training on measures of muscle strength, power, and speed in young athletes. In terms of strength training, significant, small-sized effects (*p* ≤ 0.001, SMD = 0.42) were found for measures of muscle power (e.g., CMJ) in adolescents and medium effects in children (*p* ≤ 0.001, SMD = 0.68). These authors concluded that strength training should be incorporated prior to power training in order to establish an adequate foundation of strength for power training activities. For instance, Chelly et al. (2009) showed significant training-induced improvements in muscle power (e.g., squat jump, five jump test) after 2 months of HRST versus an active control group in young soccer players. Thus, there is evidence that HRST-related strength gains may translate to power performances (Chelly et al., 2009). In terms of SET, Tse et al. (2005) examined the effects of an 8-week core SET program with two sessions per week in addition to regular strength and endurance training in elite rowers on measures of muscle power. Interestingly, SET did not produce any significant gains in medicine ball throw, vertical jump, and broad jump test performances. Findings from this study indicate that gains in maximal strength following HRST but not SET may partly translate to power performances in young female athletes.

Furthermore, our analysis confirmed the hypothesis that HRST is more effective to improve anaerobic endurance (i.e., 400-m run) compared with SET in young elite female rowers. This is well-in line with a study by Lawton et al. (2013) who showed a significant relationship for measures of maximal strength (leg press 5-RM, *r* = 0.63) but not muscular endurance (leg press 60 RM, *r* = 0.43) with anaerobic endurance (peak stroke-power over 15 maximal-strokes rowing ergometer) performance in elite rowers. Furthermore, Beattie et al. (2014) statistically aggregated the effects of HRST on anaerobic endurance using findings from 26 studies. The results showed that HRST improved anaerobic endurance (e.g., 30 s Wingate test) in competitive endurance athletes. Our study extends the existing body of literature in as much as it showed that HRST is more effective than SET to improve anaerobic endurance performance in young elite female rowers.

With regards to sport-specific performance (700-m rowing ergometer trial), the present findings indicate that SET compared with HRST induced significant large-sized effects on 700-m rowing ergometer performance in young elite female rowers. In general, this is in line with the literature demonstrating that strength training is effective to improve rowing performance (duManoir et al., 2007; Gallagher et al., 2010; Akça, 2014; Thiele et al., 2020). Previously, Ebben et al. (2004) examined the effects of an 8-weeks HRST versus SET on sport-specific performance in recreational and sub-elite female rowers with a mean age of 20 years. The authors showed that training-induced performance changes were not significantly different between HRST and SET group. Nevertheless, a trend toward larger performance gains was observed for SET (Ebben et al., 2004). Further, Lawton et al. (2013) showed that upper limbs strength endurance (30-RM seated arm pulling) is a significant predictor (*r* = −0.59) of 500-m rowing ergometer test performance in elite rowers. In addition, Jürimäe et al. (2010) showed a significant relation between lower limbs strength endurance (50% of 1-RM 7 min leg press) but not lower limbs maximal strength (1-RM leg press) with sport-specific performance (2,000-m rowing ergometer trial) in male sub-elite rowers (*r* = −0.677). With reference to the aforementioned findings from the literature, it seems plausible to argue that the observed effects of SET on rowing performance in this study can be explained by the principle of training specificity. Indeed, there is evidence that the physiological demands during SET are similar to those during rowing performance (e.g., mean and maximal heart rate, ratings of perceived exertion, and blood lactate) in male sub-elite rowers (Jürimäe et al., 2010). Taken together, it seems that SET is more effective than HRST to improve sport-specific performance in young elite female rowers.

For the secondary outcomes (e.g., dynamic balance), strength training induced significant and small-sized effects on dynamic balance (e.g., Y-balance) in young elite female rowers. Previously, beneficial effects of a 6-weeks strength training (i.e., SET) program with two sessions per week were found for dynamic balance (e.g., Y-balance test) in adolescents (Granacher et al., 2014) and following one soccer season in young elite female athletes (Lesinski et al., 2020). Thus, the results of our analysis are in line with the literature with regards to SET in young elite female rowers. Furthermore, our findings confirmed similar effects of HRST on dynamic balance compared with SET in young elite female rowers.

With regards to CoD speed, HRST induced significantly medium-sized gains, while SET did not show any improvements. Keiner et al. (2014) investigated correlations between CoD speed and 1-RM in front and back squats in adolescent soccer players aged 13 to 18 years. Interestingly, these authors reported significant moderate-to-high correlations (*r* = −0.388 to −0.697) between measures of maximal strength (e.g., 1-RM) and CoD speed. Thus, it seems that enhancements in maximal strength (e.g., 1-RM) can be transferred to improvements in CoD speed (Chaabene et al., 2020). In fact, McBride et al. (2002) investigated the effects of 8 weeks HRST (intensity: 80% of 1-RM) versus SET (30% of 1-RM) on the development of strength, power, and speed in men. These authors found significant improvements in CoD speed, with non-significant differences between the strength training types. In addition, both groups similarly improved their squat performance (1-RM). This study has a few limitations that warrant discussion. The first limitation is the low number of athletes who participated in this study. However, our *a priori* power analysis revealed that 24 participants would be sufficient to observe a large-sized group × time interaction effect for sport-specific performance. Nevertheless, future high-quality interventions are needed with larger cohorts to clarify the effects of HRST versus SET in young rowers. Second, we acknowledge that no active (i.e., rowing training only) or passive control group (no training) was included in this study, which represents a limitation when interpreting our findings especially for the influence of growth. Yet, the inclusion of a passive control group is impossible in an athletic setting because we cannot expect athletes to refrain from training for 9 weeks. Additionally, the inclusion of an active control group is hardly feasible in this specific case because strength training is an essential component of young rowers’ regular conditioning program (McNeely et al., 2005). Consequently, we cannot expect elite young rowers to conduct rowing training without performing strength training for 9 weeks. Third, due to the limited number of young rowers available at the elite sport school, we used a non-randomized controlled trial conducted across two consecutive seasons (i.e., sequential design). The non-randomized recruitment process may have contributed to the different growth rates throughout the intervention period observed in HRST and SET groups. However, growth-related effects on changes in physical fitness and sport-specific performance were removed from the analysis by including standing body height changes as a covariate in the statistical model.

In order to evaluate the effects of strength training on measures of physical fitness in recreational, elite young rowers, future research should consider the following study characteristics: (i) randomized controlled trials with one control and one strength training group. Both experimental groups should apply similar training volumes. (ii) Investigations with male and female young rowers, and (iii) researchers should focus on the most effective training types (e.g., free weight training vs machine-based training) to enhance maximal strength and/or performance in young athletes.

## Practical Applications

The aim of this study was to examine the effects of HRST versus SET in addition to regular rowing training on primary (e.g., muscle strength, muscle power, anaerobic endurance, and sport-specific performance) and secondary outcomes (i.e., dynamic balance and CoD speed) in young elite female rowers. Our analysis showed that HRST was more effective to enhance maximal strength (i.e., 1-RM bench pull and knee extension), muscle power (i.e., medicine ball push and triple hop), anaerobic endurance (i.e., 400 m run), and CoD speed (i.e., multistage shuttle run) compared with SET. In addition, statistical analysis showed that SET was more effective to increase sport-specific performance (i.e., 700-m rowing ergometer trial) compared with HRST. Furthermore, we found that power performances (e.g., DJ contact time) declined following SET while power performances did not change following HRST.

Due to the rather low number of participants (only females) in this study, further high-quality research (i.e., RCTs or CTs) is needed with young rowers. With reference to the findings of this study, coaches are advised to regularly implement HRST (four sets, 12 repetitions per set at 75–95% of 1-RM) in the training regime of young female rowers to lay a foundation of maximal strength, muscle power, anaerobic endurance, and Cod speed for subsequent sport-specific performance. Accordingly, SET (four sets, 30 repetitions per set at 50–60% of the 1-RM) should be applied to specifically induce gains in rowing performance in young female rowers.

## Data Availability Statement

The raw data supporting the conclusions of this article will be made available by the authors, without undue reservation.

## Ethics Statement

The studies involving human participants were reviewed and approved by University of Potsdam: submission No. 5/2014. Written informed consent to participate in this study was provided by the participants’ legal guardian/next of kin.

## Author Contributions

DT, OP, and UG analyzed and interpreted the data as well as wrote the original draft of the manuscript. ML critically reviewed and edited the manuscript. All authors read and approved the final manuscript.

## Conflict of Interest

The authors declare that the research was conducted in the absence of any commercial or financial relationships that could be construed as a potential conflict of interest.

## Footnotes

1 https://uk.inbody.com/
